# *In Vivo* Biodistribution and Anti-Tumor Efficacy Evaluation of Doxorubicin and Paclitaxel-Loaded Pluronic Micelles Decorated with c(RGDyK) Peptide

**DOI:** 10.1371/journal.pone.0149952

**Published:** 2016-03-01

**Authors:** Yanzuo Chen, Wei Zhang, Yukun Huang, Feng Gao, Xiaoling Fang

**Affiliations:** 1 Department of Pharmaceutics, School of Pharmacy, East China University of Science and Technology, Shanghai, 200237, China; 2 Key Laboratory of Smart Drug Delivery, Ministry of Education & PLA, School of Pharmacy, Fudan University, Shanghai, 201203, China; 3 State Key Laboratory of Molecular Engineering of Polymers, Department of Macromolecular Science, Fudan University, Shanghai, 200433, China; 4 CONRAD, Department of Obstetrics and Gynecology, Eastern Virginia Medical School, Arlington, Virginia, 22209, United States of America; University of South Alabama Mitchell Cancer Institute, UNITED STATES

## Abstract

The treatment of squamous carcinoma, especially multidrug resistance (MDR) tumors, represents one of the most formidable challenges in oncology. In this study, integrin-mediated Pluronic-based micellar system (c(RGDyK)-FP-DP) was proposed as a drug delivery system to enhance the *in vivo* anti-tumor efficacy in MDR human squamous carcinoma (KBv)-bearing. Following the recognition by integrin proteins express on the cell surface, cellular uptake and *in vitro* anti-tumor efficacy of c(RGDyK)-FP-DP were better than conventional PF-DP in KBv cells. The tumor homing specificity and further *in vivo* anticancer efficacy of c(RGDyK)-FP-DP were performed using subcutaneous KBv tumor-bearing mice model, respectively. Compared with PF-DP, c(RGDyK)-FP-DP demonstrated more drug accumulation in tumor and relatively less drug accumulation in heart, and an extended median survival time in the KBv tumor-bearing mice model. Furthermore, preliminary *in vivo* subacute toxicity evaluation was also conducted by the measurement of histopathology, blood cell counts and clinical biochemistry parameters. Results showed that no obvious toxicity was observed to the hematological system or heart after a series of intravenous administration of c(RGDyK)-FP-DP. In conclusion, our results suggested that c(RGDyK) peptide conjugated Pluronic micelles could be a promising vehicle for enhancing the treatment of MDR human squamous carcinoma.

## 1. Introduction

Oral squamous cell carcinoma (OSCC), accounts for the majority of head and neck cancers, is one of the most common cancers in the world [1, 2] with a five-year relative survival rate less than 35% in advanced stage at initial diagnosis.[3] Delayed diagnosis, cancer recurrence, metastasis and resistant to treatment may attribute to this poor survival rate.[4] Oral cancer arises from the neoplasms of the oral cavity with more than 90% of cases classified as squamous cell carcinomas. Conventional treatments have limited efficacy and result in adverse systemic and cytotoxic effects on normal cells.[5] Appropriate chemotherapy with low toxicity and targeting important pathways involved in cancer development simultaneously is a promising strategy for oral cancer treatment.[6]

Based on histopathology during the tumor development, the vascular supply is no longer adequate to support the increasing metabolic demands of the rapidly proliferating tumor cells, and then induces the up-regulation of vasoactive endothelial growth factor and the promotion of new blood vessel formation from the existing vasculature. These new formed vessels often lack the complex structure of the normal vasculature and result in the endothelial permeability.[7] The integrin α_v_β_3_ as an important biomarker, is known for its role in cancer progression and over-expressing in most tumor cells and sprouting tumor vessels.[8] It has been demonstrated that RGD (arginine-glycine-aspartic acid) short peptides can specifically bind with integrin α_v_β_3_ and play a significant role in regulating tumor growth, metastasis and angiogenesis.[9, 10] The high affinity interaction between cancer-related integrins and RGD peptides has led to the widely use of RGD peptide as a ligand for specific integrin-targeted drug and gene delivery applications.[11, 12]

In our previous studies, we have successfully constructed a cyclic RGD peptide functionalized Pluronic-based nanomicellar system encapsulating doxorubicin (DOX) and paclitaxel (PTX) (c(RGDyK)-FP-DP) intended for antiangiogenesis and drug resistance modulation in multidrug resistance (MDR) cancer cells. [13] Simultaneous administration of DOX and PTX to patients with metastatic cancer is superior to that of individual drug therapy in terms of tumor regression rates.[14, 15] Unfortunately, both DOX and PTX are found to be the substrate of P-glycoprotein (P-gp), multidrug resistance-associated protein (MRP) and breast cancer resistant protein (BCRP),[16–19] which can pump the cytotoxic drugs out of the tumor cells and thus lower the intracellular concentration of therapeutic agents.[20] We found that Pluronic-based polymeric micelles have shown abilities to enhance cytotoxicity of anticancer agents against MDR cancer cells, prolong blood circulation time and modify the biodistribution behavior of the anticancer drug.[21–26]

As demonstrated, c(RGDyK) peptide functionalized nanomicellar system can overcome the drawbacks of low transport of chemotherapeutics across the blood-tumor barrier (BTB) in MDR cancer cells using *in vitro* cell-based models.[13] However, most of OSCC are solid tumors, and there are hypoxic and avascular tumor regions distant from the vascular bed, quite different from the *in vitro* situation, and these chemotherapy “blind areas” ineluctably lead to the relapse of tumor.[27, 28] Thus, the *in vivo* biodistribution, anti-tumor efficacy and basic safety testing of c(RGDyK)-FP-DP were fully conducted in MDR OSCC-bearing mice model in this study, aiming to identify whether c(RGDyK)-FP-DP is a relatively safe and efficacious nanodrug delivery system for the treatment of MDR OSCC.

## 2. Materials and Methods

### 2.1. Materials and animals

Pluronic P105 and F127 were kindly provided by BASF Ltd. (Shanghai, China). P105-DOX and c(RGDyK)-F127 were synthesized in our lab.[13, 29] Doxorubicin (DOX) was obtained from Beijing Huafeng United Technology Co. Ltd. (Beijing, China), and Paclitaxel (PTX) was purchased from Xi’an Sanjiang Bio-Engineering Co. Ltd. (Xi’an, China). Free PTX solution was prepared according to the commercial formulation of Taxol. Dioctadecyl-3, 3, 3′, 3′-tetramethylindotricarbocyanine iodide (DIR) was purchased from Biotium (Life Technologies, Carlsbad, CA, USA). Hoechst 33258 staining kit was purchased from Beyotime Biotechnology Co., Ltd. (Nantong, China). Purified deionized water was prepared by the Milli-Q plus system (Millipore Co., Billerica, MA, USA). All other reagents and chemicals were of analytical grade and were used without further purification.

The multidrug resistant oral squamous cell carcinoma KBv cell line was purchased from Nanjing KeyGen Biotech. Co. Ltd. (Nanjing, China), with 0.2 μg/mL of vinblastine in RPMI-1640 medium to maintain the drug resistance for KBv. KBv cells were cultured in medium supplemented with 10% FBS, 100 IU/mL penicillin and 100 μg/mL streptomycin sulfate at 37°C with 5% CO_2_ under fully humidified conditions.

Male BALB/c nude mice (20± 2) g, supplied by Department of Experimental Animals, Fudan University (Shanghai, China), were acclimated at 25°C and 55% of humidity under natural light/dark conditions for 1 week before the proposed experiments. All animal studies were specifically approved by the ethics committee of Fudan University (Shanghai, China) and carried out in accordance with guidelines evaluated and approved by this committee. All animal surgery was performed under sodium pentobarbital anesthesia (60 mg/kg, i.p.), and all efforts were made to minimize suffering.

### 2.2. Preparation of micellar system

c(RGDyK)-FP-DP was prepared by the thin-film hydration technique described previously.[23] Briefly, F127 (22.2 mg), c(RGDyK)-F127 (11.1 mg), P105-DOX (36.7 mg), P105 (230 mg) and PTX (6 mg) were dissolved in 5 mL dichloromethane. A thin polymeric film was formed in a round-bottom flask by removing the organic solvents from the mixed solution aforementioned through rotary evaporation at 37°C. Then, the dry polymeric film was hydrated with deionized water, which was then filtrated through 0.22 μm membrane, followed by lyophilization. Pluronic micelles without c(RGDyK) decoration (PF-DP) was also prepared as above, without the addition of c(RGDyK)-F127, instead using of equivalent dose of F127. HPLC was used to measure the drug content of DOX and PTX.[13]

The preparation of DIR-labeled mixed micelles (PF/DIR and c(RGDyK)-FP/DIR) were performed in a similar way, except that 80 μL DIR (1 mg/mL stock solution in dichloromethane) was added into the mixture of methanol and Pluronic block polymers before the removal of the organic solvents. After preparation, the free DIR was removed via CL-4B column chromatography (Hanhong Chemica Co. Ltd., China).[30]

### 2.3. *In vitro* cellular uptake study

The cellular uptake measurement was performed via fluorescent microscopy, HPLC and BCA method. For qualitative analysis, KBv cells were seeded at a density of 1×10^4^ cells/well in 24-well plates and incubated for 24 h. Cells were incubated with PF-DP or c(RGDyK)-FP-DP at a carrier concentration of 100 μg/mL for 4 h at 37°C. The solution was removed and the cells were washed three times with ice-cold PBS (pH 7.4) and then visualized under fluorescent microscope (Leica DMI 4000B, Germany). To observe if c(RGDyK) peptide could hinder the receptor-mediated endocytosis, KBv cells were pre-incubated with 0.3 μg/mL of free c(RGDyK) peptide for 1 h before exposure to PF-DP or c(RGDyK)-FP-DP.

For quantitative study, 24-well cell culture plates were seeded with 1×10^5^ KBv cells per well and incubated at 37°C for 24 h to allow the cell attachment. After 24 h incubation, the medium was replaced by 5 μg/mL of PF-DP or c(RGDyK)-FP-DP in FBS-free DMEM. After 4 h incubation at 37°C, cells were washed with cold PBS twice and then lysed with 0.4 mL PBS containing 1% Triton X-100. After incubation, 100 μL cell lysate was withdrawn and extracted with methanol (200 μL/sample), then the mixture was subjected to probe-type ultrasonic treatment (400 W, 10 cycles with 2 s active-3 s duration, JY92-II, Scientz 238 Biotechnology Co., Ltd., China) in ice bath. After extraction, the mixture was centrifuged at 5000 rpm for 5 min, and the supernatant was analyzed by HPLC. The protein content in the sample was determined using the BCA protein assay kit in accordance with the method specified by the manufacturer. Cellular accumulation of DOX and PTX was normalized with respect to the total protein content determined by the BCA method.

### 2.4. *In vitro* anti-tumor activity evaluation

KBv cells were seeded in 96-well plates at the density of 1×10^4^ cells/well. After 24 h incubation at 37°C with 5% CO_2_, the medium was removed, and the cells were incubated for another 72 h with the media containing DOX, PTX, mixture of DOX and PTX (DOX+PTX), PF-DP or c(RGDyK)-FP-DP of at various drug concentrations. Cell survival was measured using MTT assay.[23] The absorbance at 570 nm of each well was measured by a microplate reader (Tecan Safire2, Switzerland).

### 2.5. Biodistribution studies

Tumor-bearing mice model was established by inoculating 0.2 mL PBS containing 5×10^6^ KBv cells into right hind leg of the mice, and the tumor was allowed to grow for approximately 1 cm^3^ in diameter. *In vivo* real-time fluorescence imaging analysis was used to qualitatively evaluate the tissue distribution of Pluronic-based polymeric micelles. Tumor-bearing mice were injected with 100 μL DIR-labeled Pluronic mixed micelles (DIR content: 0.2%) via the tail vein. The anesthetized mice were placed on a heated plate (37°C). The fluorescent scans were performed at various time points (2, 6, 12, 24 and 48 h) post i.v. using the Maestro™ *in vivo* imaging system (excitation: 700–950 nm, emission: 780 nm long-pass; Cambridge Research and Instrumentation Inc, Woburn, MA, USA). The tumor bearing mice were then sacrificed at 48 h post-injection and the tumor and major organs including heart, liver, spleen, lung and kidney were harvested. Each organ was rinsed with PBS and the fluorescence intensity was detected.

In order to quantify the amount of DOX and PTX in tissues, tumor-bearing mice were divided into three groups at random, and injected with 0.1 mL of DOX+PTX, PF-DP or c(RGDyK)-FP-DP intravenously via the tail vein (total drug: 5 mg/kg), respectively. Then, at specific time points (0.5, 1, 3, 5, 9, 24 and 48 h) post-injection, three mice from each group were sacrificed after drawing blood (0.5 mL) from the periorbital area. Blood was immediately centrifuged at 1000 rpm for 10 min to obtain the plasma, which was then stored at -70°C prior to HPLC analysis. Major organs were excised, washed with cold saline, dried on filter paper, weighed, and stored at -70°C until further analysis by HPLC. The tissues were homogenized in a mixture of acetonitrile and water (50: 50, v/v) before extraction. DOX and PTX in plasma and tissue samples were performed by liquid-liquid extraction respectively, as reported before,[7, 13, 31] prior to HPLC analysis.

### 2.6. *In vivo* antitumor efficacy

*In vivo* anti-tumor activity was evaluated using subcutaneous KBv tumor-bearing BALB/c nude mice, which was established by inoculating 0.2 mL PBS containing 2×10^6^ KBv cells subcutaneously in the right hind leg of the mice. The first dose was given to the mice when the tumor volume reached about 50–100 mm^3^ (designated as day 0). The mice were randomized into four groups and treated with 0.1 mL of saline, DOX and PTX mixture (DOX+PTX, DOX: PTX = 2: 3, w/w), PF-DP and c(RGDyK)-FP-DP at a 10 mg/kg dose of total drug via tail vein injection on day 0, 4 and 8, respectively. Tumor size was monitored via caliper measurement every other day and the tumor volume was estimated using the Eq (1):

$$V=0.5\times \mathrm{length}\times {\left(\mathrm{width}\right)}^{2}$$ (1)

On day 14, the mice were sacrificed by cervical dislocation, and the tumor mass was harvested, weighed and photographed. The inhibitory rate of tumor (IRT%) was calculated according to Eq (2):

$$IRT\%=\frac{W_{\mathrm{control}}-W_{\mathrm{treated}}}{W_{\mathrm{control}}}\times 100\%$$ (2)

As for the survival study, a total of 48 KBv tumor-bearing mice were randomized into 6 groups: saline, DOX, PTX, DOX+PTX, PF-DP and c(RGDyK)-FP-DP. The dosage regimen was the same as above, and health and survival of mice were monitored daily. Mice that were unable to right themselves within 20 seconds were euthanized immediately and recorded as dead for the purpose of survival study. Animals were euthanized by CO_2_ asphyxiation and death was confirmed by verifying respiratory arrest followed by cervical dislocation. The mortality aspects of the protocol of survival study was specifically reviewed and approved by the ethics committee of Fudan University (Shanghai, China).

### 2.7. Safety evaluation

For safety evaluation of c(RGDyK)-FP-DP, the body weight of each mouse was determined every other day. Heart and tumor tissue samples were processed routinely into paraffin on day 9 and day 14, respectively, sectioned at a thickness of 5 μm followed by hematoxylin and eosin (H&E) staining for histopathological analysis, and then visualized under microscope. Moreover, heart and tumor tissues were frozen in optimal cutting temperature embedding medium (Sakura, Torrance, CA, USA) at -80°C. Frozen sections of 20 μm thickness were prepared and stained with Hochest 33258 kit according to the specification. After washed with PBS, the sections were immediately examined under the fluorescent microscope.

Blood was collected in tubes containing EDTA-2K and a portion of blood sample as used for the measurement of white blood cell (WBC), red blood cell (RBC), platelets. In order to collect the serum, the rest of the blood sample was allowed to stand for 30 min at room temperature and then centrifuged at 3000 rpm for 15 min at 4°C and the aspartate transaminase (AST) and creatine kinase (CK) levels were tested. All the blood and serum measurements were conducted at the Shanghai Institute for Food and Drug Control.

### 2.8. Data analysis

Data were expressed as mean ± standard deviation (SD). Multiple comparisons were performed using ANOVA. Survival was analyzed using the Kaplan-Meier method and log-rank test. A value of P < 0.05 was considered to be significant.

## 3. Results and Discussion

### 3.1. *In vitro* cellular uptake studies

**Fig 1:**
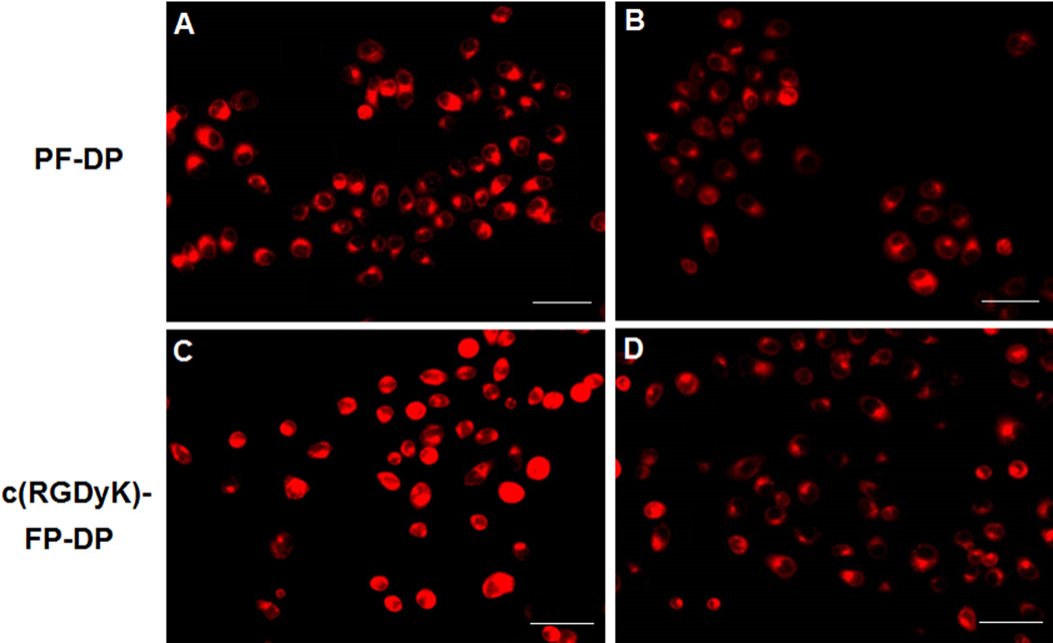
The qualitative analysis for KBv cells cellular uptake. Celluar uptake studies in KBv cells after 1 h treatment with (A) PF-DP and (C) c(RGDyK)-FP-DP or 1 h pre-incubation with 0.3 μg/mL of free c(RGDyK) peptide and then exposure to (B) PF-DP and (D) c(RGDyK)-FP-DP. Cells were examined by fluorescent microscopy; Red: DOX; Bar: 30 μm.

The conjugated DOX in polymeric micelles was used as the fluorescence probe to trace the micelles in KBv cells. Results were shown qualitatively by the fluorescent images (Fig 1A and 1C). KBv cells treated with c(RGDyK)-FP-DP exhibited higher red fluorescence intensity than that of PF-DP, suggesting that c(RGDyK) decorated on the surface of micelles could facilitate the cellular uptake of polymeric micelles in KBv cells. Additionally, the uptake of c(RGDyK)-modified micelles could be competitively inhibited by free c(RGDyK) peptide (Fig 1B and 1D), which further proved that the uptake of c(RGDyK)-conjugated micelles was at least in part mediated by the interaction of c(RGDyK) and integrin proteins expressed on the surface of KBv cells. Precondition with c(RGDyK) peptide, a ligand of integrin, the cellular uptake of c(RGDyK)-FP-DP was reduced probably due to the fact that integrin receptor expressed in KBv cells could competitively bind with the free ligands. In this work, KBv cell line was selected as the cell model, acting as the classical OSCC with MDR phenomenon.[22, 26, 32] Although integrin was not richly expressed in KB cells as reported,[33, 34] in our previous study it was found that DOX and PTX cellular uptake of c(RGDyK)-FP-DP were higher than those of PF-DP in KBv cells (P< 0.01). DOX and PTX concentration of c(RGDyK)-FP-DP in KBv cells were 360 and 433 ng/mg protein, respectively, exhibited 1.29 and 1.31 times higher cellular uptake than those of PF-DP.

In addition, as reported[35] the drug intracellular accumulation of Pluronic polymeric micelles was comparable to that of P-gp inhibitor (CsA) group (P< 0.05) in MCF-7/ADR cells, suggesting that Pluronic P105/F127 based polymeric micelles were able to enhance drug accumulation in MDR tumor cells due to the presence of Pluronic unimers, which have been identified as the biological modifiers.[36–39] However, further experiments need to be conducted to test the difference in integrin receptor expression between KB and KBv cells.

### 3.2. *In vitro* anti-tumor efficacy evaluation

*In vitro* anti-tumor efficacy was measured in KBv cells after incubation with DOX, PTX, DOX+PTX, PF-DP or c(RGDyK)-FP-DP for 72 h. The cell viability was evaluated using MTT assay. The IC_50_ values were found to be 16.422± 2.691 μg/mL for DOX, 8.232± 0.750 μg/mL for PTX, 7.121± 0.533 μg/mL for DOX+PTX, 0.802± 0.136 μg/mL for PF-DP and 0.146± 0.073 μg/mL for c(RGDyK)-FP-DP, respectively. The results showed that both mixed micelles exhibited strong inhibitory effects to the proliferation of KBv cells, compared with free drug mixtures. Thus, it was suggested that Pluronic micelles may act as a chemosensitizer and potentiate cytotoxic effects in KBv cancerous cells, which was in good agreement with previously reported results.[13, 22] Furthermore, it was found that *in vitro* anti-tumor efficacy of Pluronic-based micelles decorated with c(RGDyK) (c(RGDyK)-FP-DP) significantly increased as compared with PF-DP, indicating that the decorating c(RGDyK) peptide might facilitate c(RGDyK)-FP-DP uptake in KBv cells which may further enhance the cytotoxicity due to the potential increased intracellular concentration of drugs.

### 3.3. Biodistribution studies

**Fig 2:**
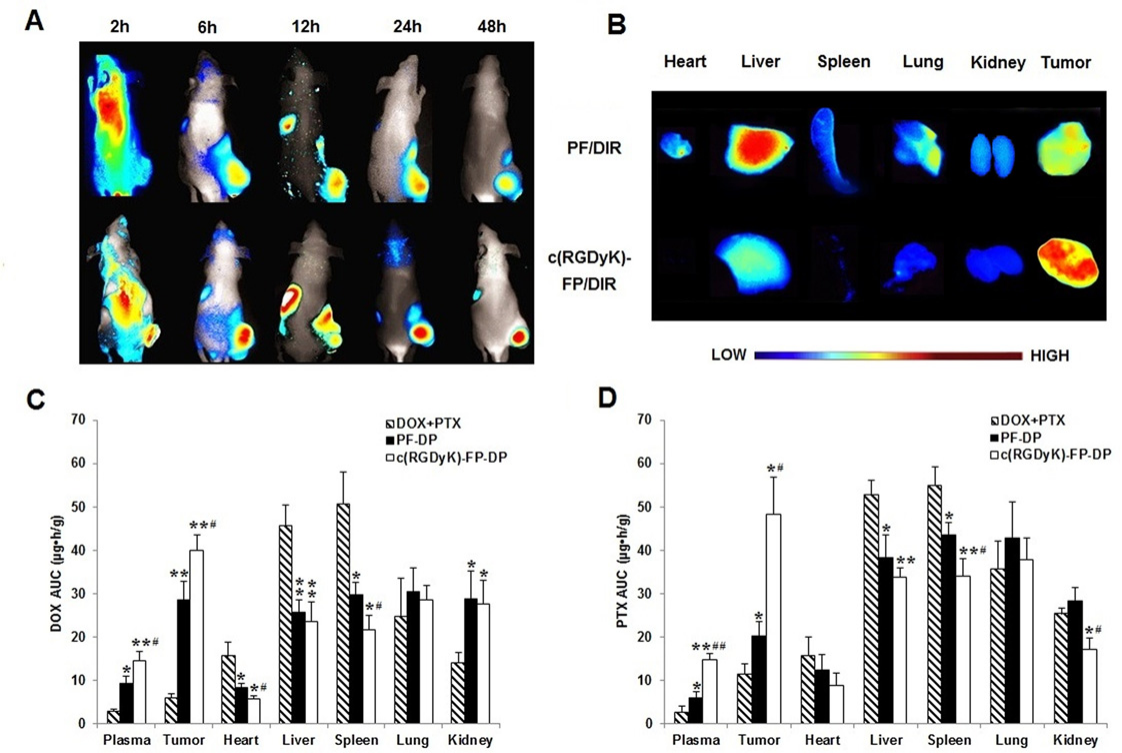
Biodistribution studies in tumor bearing mice. (A) *In vivo* fluorescence imaging of subcutaneous KBv tumor-bearing BALB/c nude mice after i.v. injection of PF/DIR and c(RGDyK)-FP/DIR at different time points; (B) Images of dissected organs of tumor bearing mice sacrificed at 48 h after i.v. injection of PF/DIR and c(RGDyK)-FP/DIR; The AUC_0→48h_ of (C) DOX and (D) PTX in plasma and tissues after i.v. administration of the physical mixture of DOX and PTX (DOX+PTX), PF-DP and c(RGDyK)-FP-DP to subcutaneous KBv tumor-bearing BALB/c nude mice at a single 5 mg/kg dose of total drug, respectively. *P< 0.05, **P< 0.01, compared with DOX+PTX; ^#^P< 0.05, ^##^P< 0.01, compared with PF-DP. Mean ± standard deviation (n = 3).

*In vivo* multispectral fluorescent imaging analysis was used to evaluate the targeting effect and time-dependent biodistribution of different DIR-labeled Pluronic-based polymeric micelles in live subcutaneous KBv tumor-bearing nude mice after i.v. administration through the tail vein (Fig 2A). The fluorescence detected in the tumor indicated that both Pluronic-based polymeric micelles can contribute to tumor accumulation via EPR effect. However, compared with PF/DIR group, the NIR fluorescence intensity in the tumor region of c(RGDyK)-FP/DIR group was much higher throughout the study, suggesting the active targeting effect of c(RGDyK)-FP/DIR *ex vivo* fluorescence evaluation of dissected organs (heart, liver, spleen, lung and kidney) and tumor tissue at 48 h post-injection also confirmed that the tumor accumulation of c(RGDyK)-FP/DIR was much more than that of PF/DIR. We also observed that the fluorescence signal in liver and spleen of c(RGDyK)-FP/DIR were lower than that of PF/DIR micelles (Fig 2B).

**Fig 3:**
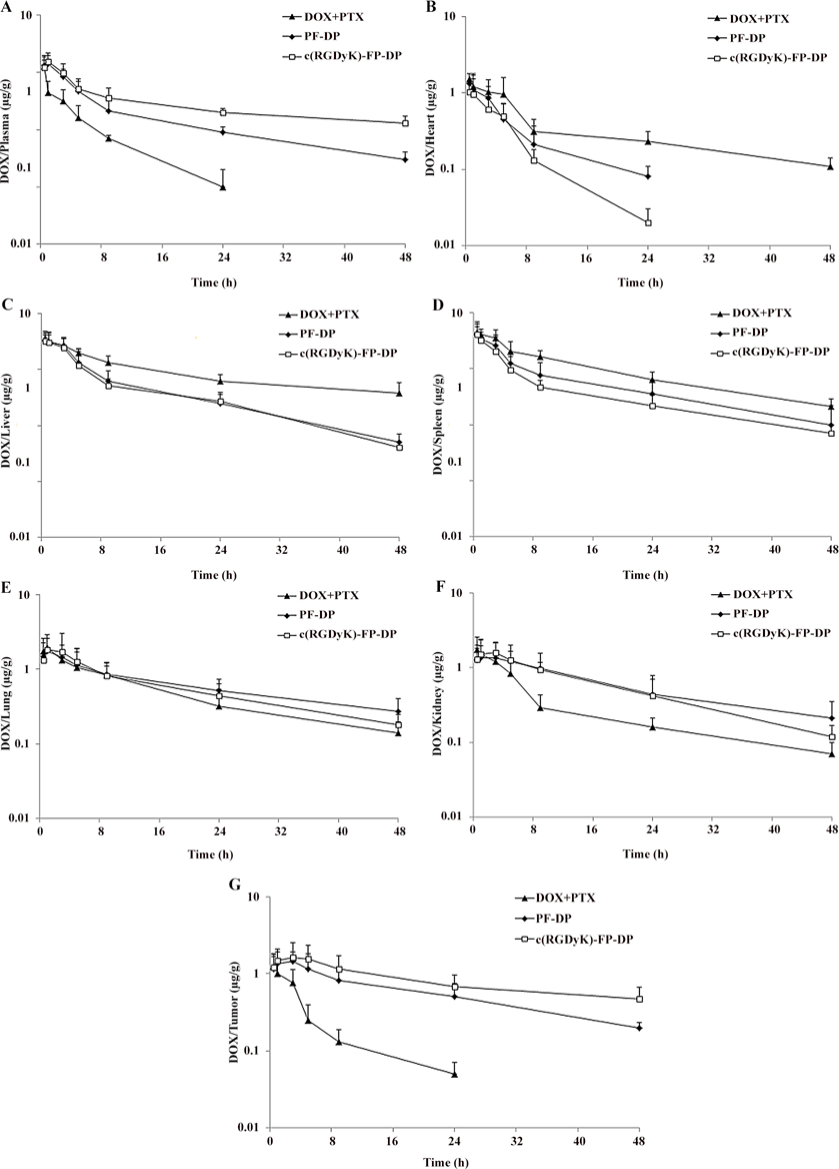
The *in vivo* DOX concentration of free DOX and PTX mixture (DOX+PTX), PF-DP and c(RGDyK)-FP-DP in plasma and different tissues at specific time points following i.v. administration to KBv tumor-bearing mice at a single 5 mg/kg dose. (A) Plasma; (B) Heart; (C) Liver; (D) Spleen; (E) Lung; (F) Kidney; (G) Tumor. Mean ± standard deviation (n = 3).

**Fig 4:**
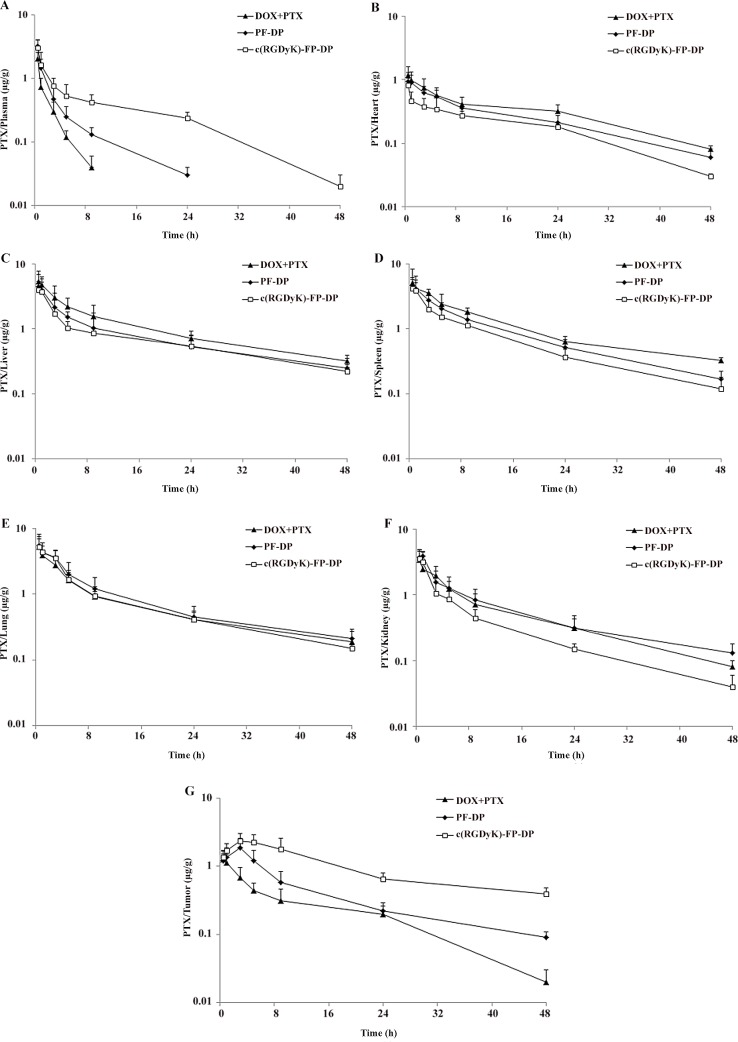
The *in vivo* PTX concentration of free DOX and PTX mixture (DOX+PTX), PF-DP and c(RGDyK)-FP-DP in plasma and different tissues at specific time points following i.v. administration to KBv tumor-bearing mice at a single 5 mg/kg dose. (A) Plasma; (B) Heart; (C) Liver; (D) Spleen; (E) Lung; (F) Kidney; (G) Tumor. Mean ± standard deviation (n = 3).

DOX and PTX concentrations in blood, heart, liver, spleen, lung, kidney and tumor tissues were also measured after i.v. administration of DOX+PTX, PF-DP or c(RGDyK)-FP-DP (Figs 3 and 4), and the calculated AUC_0→48h_ of DOX and PTX in tissues and plasma were shown in Fig 2C and 2D. The AUC_0→48h_ in tissues for DOX was decreased in the following order: spleen > liver > lung > heart > kidney > tumor > plasma while the corresponding order for PF-DP and c(RGDyK)-FP-DP were lung > spleen > kidney > tumor > liver > plasma > heart and tumor > lung > kidney > liver >spleen > plasma > heart, respectively. The DOX AUC_0→48h_ of mixed micelles groups was higher in plasma, tumor and kidney, lower in heart, liver and spleen compared with DOX+PTX group (P< 0.05), while the DOX AUC_0→48h_ of c(RGDyK)-FP-DP was higher in plasma and tumor, lower in heart and spleen compared with PF-DP (P< 0.05). Similarly, the PTX AUC_0→48h_ of mixed micelles groups was higher in plasma and tumor, lower in liver and spleen compared with DOX+PTX group (P< 0.05), while the PTX AUC_0→48h_ of c(RGDyK)-FP-DP was higher in plasma and tumor, lower in spleen and kidney compared with PF-DP (P< 0.05). Moreover, the amount of DOX and PTX in tumor tissues treated with c(RGDyK)-FP-DP was 1.40 and 2.39 times higher than that of PF-DP (P < 0.05), respectively.

An enhanced permeability and retention (EPR) effect is likely to be achieved if micelles could circulate *in vivo* for a sufficient time period to allow the anti-cancer drugs released in the tumor tissues. In this study, results indicated that c(RGDyK)-FP-DP could significantly increase drug accumulation in tumor and decrease non-specific uptake in reticuloendothelial systems (RES). The particle size of c(RGDyK)-FP-DP was around 23 nm, which could perfectly facilitate the extravasation from leaky capillaries,[40] and the PEO hydrophilic shell of c(RGDyK)-FP-DP can efficiently decrease the adsorption of plasma protein to micelles *in vivo* which may diminish the elimination by RES, resulting in the prolonged circulation time *in vivo*. Additionally, c(RGDyK) is a peptide that issued for neovasculature targeting delivery since its high binding efficiency with α_v_β_3_ which is overexpressed on neovascular endothelial cells.[8, 11] Thus, c(RGDyK)-FP-DP also has potential to cut off the metabolic nutriment supply during angiogenesis in tumor.

### 3.4. *In vivo* anti-tumor efficacy

**Fig 5:**
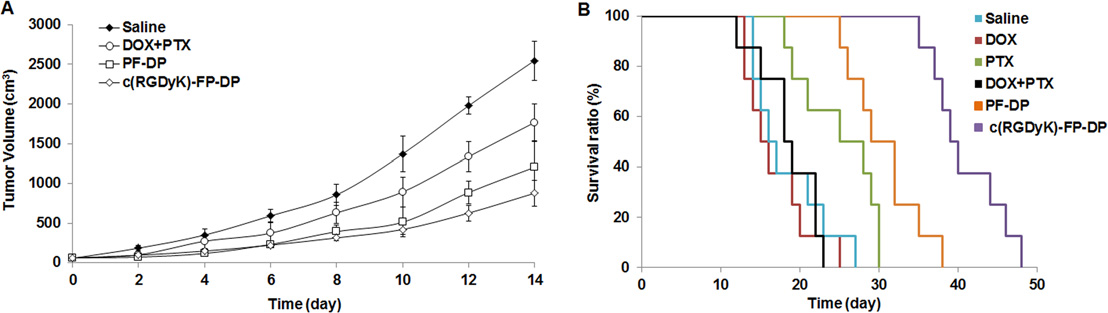
*In vivo* anti-tumor efficacy of c(RGDyK)-FP-DP in subcutaneous KBv tumor-bearing mice. (A) Changes in the tumor volume after treatments. Mean ± standard deviation (n = 6); (B) Kaplan-Meier survival curves of KBv tumor-bearing mice treated with different DOX+PTX, PF-DP and c(RGDyK)-FP-DP (10 mg/kg total drug for each dosing). Mean ± standard deviation (n = 8).

The excised tumors were weighed and the relative tumor volume measured of all the treatment groups was smaller than that of saline group throughout the experimental period (Fig 5A). The inhibitory rates of tumor (IRT) treated by DOX+PTX, PF-DP and c(RGDyK)-FP-DP on day 14 were 40.0%, 57.7% and 88.4%, respectively. The antitumor efficacy of c(RGDyK)-FP-DP was found to be superior to that of DOX+PTX and PF-DP in subcutaneous KBv tumor mice model (P< 0.001). The *in vivo* antitumor efficacy result was consistent with that of *in vitro* cytotoxicity *tests*. The synergistic effect of enhanced cellular uptake and the neovasculature targeting could be the main reason for the significant suppression of tumor growth in c(RGDyK)-FP-DP group.

The anti-tumor effect of c(RGDyK)-FP-DP in close comparison with of the other groups including saline, DOX+PTX and PF-DP were also examined by *in vivo* survival test using squamous carcinoma subcutaneous KBv tumor-bearing mice. We can see from the Kaplan-Meier plot (Fig 3B) that the antitumor efficacy of c(RGDyK)-FP-DP was superior to the other groups. As shown in Table 1, the mean survival time (MST) of c(RGDyK)-FP-DP group, PF-DP group, DOX+PTX group, DOX group, PTX group and saline group were 40, 32, 18, 16, 25 and 16 days, respectively. The 50% of saline treated mice were died by 16 days due to rapid growth of MDR tumors. The median survival values of DOX and DOX+PTX free drug groups were 16 and 18 days, respectively. In contrast, when the mice were treated with PF-DP or c(RGDyK)-FP-DP, the median survival values were 32 and 40 days, respectively, suggesting c(RGDyK)-FP-DP exhibited the strongest antitumor activity, in terms of MST and median survival values, and the greatest increased life spans (ILS) (P< 0.001).

**Table 1:** *In vivo* efficacy of various formulations on the survival of KBv tumor-bearing mice.

Groups	MST (days)	Median (days)	ILS^a^ over saline (%)	ILS over DOX (%)	ILS over PTX (%)	ILS over DOX+PTX (%)
**Saline**	17.43 ± 4.03	16	-	-	-	-
**DOX**	19.00 ± 4.61	16	9.01	-	-	-
**PTX**	24.29 ± 4.76**^#^	25	39.4	-	-	-
**DOX+PTX**	18.17 ± 3.97^@^	18	4.25	-	-	-
**PF-DP**	30.43 ± 4.60***^###^^@^^$^	32	74.6	60.2	25.3	67.5
**c(RGDyK)-FP-DP**	40.88 ± 4.46***^###^^@@@^^$^^%^	40	134.5	115.2	68.3	125.0

**Notes:** Mean ± standard deviation (n = 8).

^a^ ILS = [(T/C-1)× 100%], where T and C denote MST of the treated and control groups, respectively.

** P< 0.01

*** P< 0.001 when compared with saline group.

^#^ P< 0.05

^###^ P< 0.001 when compared with DOX group.

^@^ P< 0.05

^@@@^P< 0.001 when compared with PTX group.

^$^ P< 0.001 when compared with DOX+PTX group.

^%^ P< 0.001 when compared with PF-DP group.

**Fig 6:**
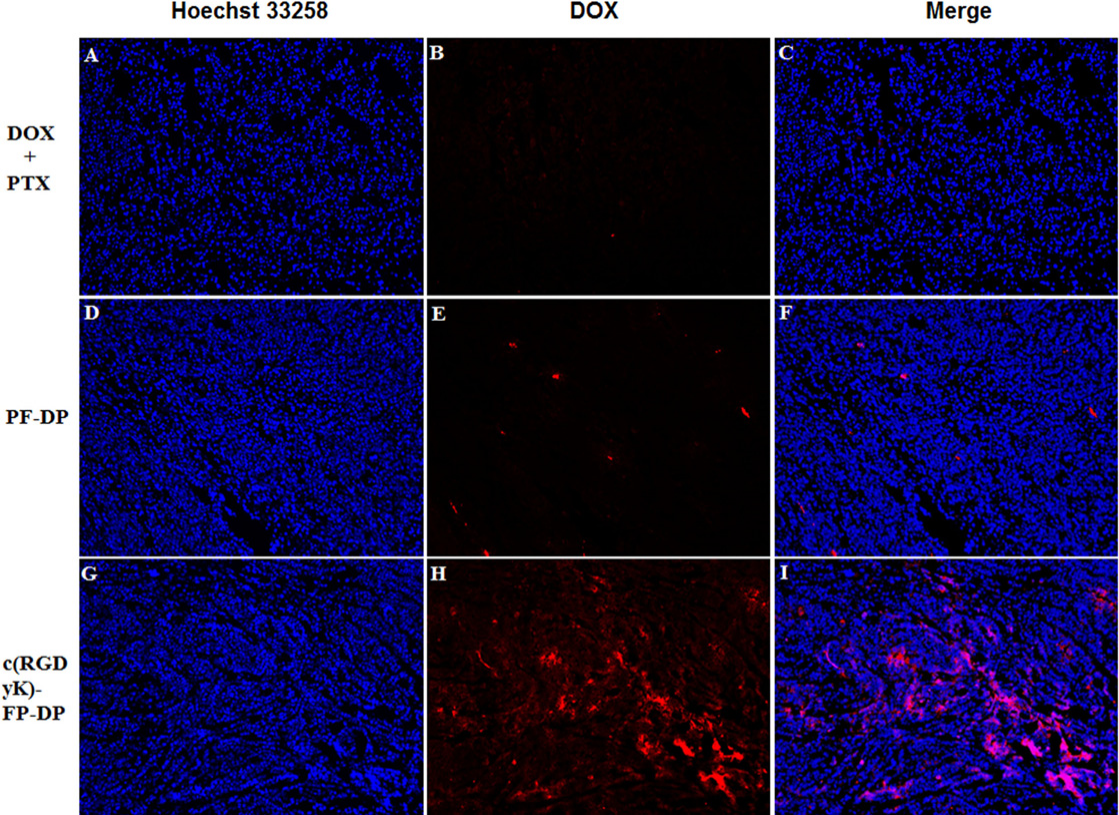
Fluorescence images of tumor sections from KBv tumor-bearing BALB/c nude mice after various systemic administration. (A, B, C) DOX+PTX; (D, E, F) PF-DP; (G, H, I) c(RGDyK)-FP-DP; Red: DOX; Blue: Hoechst 33258. Original magnification: ×20.

**Fig 7:**
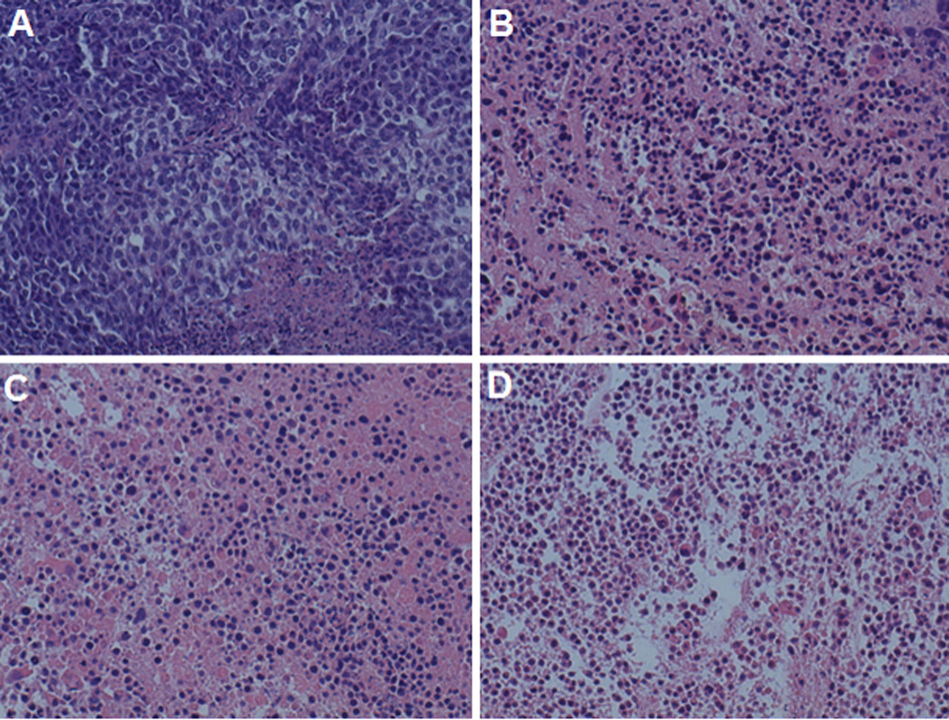
Hematoxylin and eosin (H&E) stained tumor sections isolated from mice on 14th day after various different treatments. Notes: (A) Saline, (B) DOX+PTX, (C) PF-DP and (D) c(RGDyK)-FP-DP. Original magnification: ×20.

The DOX distribution in tumor was observed qualitatively by fluorescent microscope (Fig 6). The red fluorescence intensity of DOX in DOX+PTX, PF-DP and c(RGDyK)-FP-DP group was found to be in a increasing order. The results suggested that the highest DOX amount was found in c(RGDyK)-FP-DP group which is in a good agreement with the results of biodistribution study. In addition, in order to evaluate the necrosis level in tumors among the groups treated with DOX+PTX, PF-DP, c(RGDyK)-FP-DP, and saline, the tumors were excised from the mice right after the 14 day treatments. Tumor tissues were sliced, stained by hematoxylin and eosin (H&E), and analyzed via optical microscopy. Qualitatively, the most necrosis area were observed in the tumor treated by c(RGDyK)-FP-DP-treated (Fig 7). The possible reasons were listed as followings: (1) Pluronic could promote the release of cytochrome C and accumulation of ROS in MDR cells, which may enhance the apoptosis of PF-DP and c(RGDyK)-FP-DP groups.[26, 41] (2) The increased local concentration of DOX and PTX in the tumor tissue could be due to the EPR and active integrin-mediated effects.

### 3.5. Safety evaluation

Animal body weight change was commonly used as a marker of *in vivo* safety studies. The body weight of the mice treated with DOX+PTX decreased dramatically at day 1 after the first dose, and remained declining post treatment (data not shown). However, the body weight of mice treated with micelles slightly decreased initially after dosing but recovered soon. Therefore, there was no significant body weight loss after the treatment of polymeric micelles, suggesting that a potential low systemic toxicity of PF-DP and c(RGDyK)-FP-DP (Table 2).

**Table 2:** The change in the body weight and tumor weight after different treatments in KBv tumor-bearing mice.

Groups	Body weight (g)		Tumor weight (g)	IRT (%)
	Day 0	Day 14		
**Saline**	21.78± 1.14	25.13± 2.16	0.95± 0.16	-
**DOX+PTX**	21.93± 1.70	19.44± 1.19	0.52± 0.22	45.26
**PF-DP**	22.06± 1.06	24.32± 1.68*	0.35± 0.11	63.16
**c(RGDyK)-FP-DP**	21.47± 1.78	24.18± 1.45*	0.11± 0.10*^#^	88.42

**Notes:** Mean ± standard deviation (n = 6).

* P< 0.05 when compared with DOX+PTX group.

^#^ P< 0.05 when compared with PF-DP group.

To reveal the potential side effects in hematology, basic blood biochemistry was analyzed in this study. Results showed that DOX+PTX could significantly decreased WBC and platelets in subcutaneous KBv tumor-bearing mice compared with the control group (P< 0.05) (Table 3). WBC and platelets of PF-DP and c(RGDyK)-FP-DP groups were higher than those of mice receiving DOX+PTX but comparable to saline group.

**Table 3:** The hematological results in mice after different treatments.

Groups	WBC (10^9^/L)	RBC (10^12^/L)	Platelets (10^9^/L)	AST (IU/L)	CK (IU/L)
**Saline**	9.03 ± 1.10	6.88 ± 1.19	1865.3 ± 113.8	266.2 ± 57.4	1632.9 ± 230.2
**DOX+PTX**	5.06 ± 0.73*	7.23± 1.20	1097.4 ± 141.0**	478.1 ± 47.3**	3043.7± 424.9*
**PF-DP**	8.49 ± 0.74	7.08 ± 1.25	1946.6 ± 165.1	279.4 ± 40.2	1821.4± 156.7
**c(RGDyK)-FP-DP**	9.11 ± 0.83	6.71 ±0.92	1832.1 ± 142.8	253.5± 32.6	1681.0± 194.6

**Notes:** Mean ± standard deviation (n = 3).

*P< 0.05

**P< 0.01 when compared with saline group.

As reported, DOX can trigger the disruption of cardiac myocytes and release of intracellular CK and AST into the serum.[7] In this study, serum CK and AST measurements in mice are depicted in Table 3. AST and CK of mice treated with DOX+PTX were significantly higher as compared with saline group, indicating the potential DOX associated side effects. However, there was no statistically significant difference between micelle groups and the control group, suggesting no obvious cardiac toxicity in PF-DP and c(RGDyK)-FP-DP groups (P>0.05).

**Fig 8:**
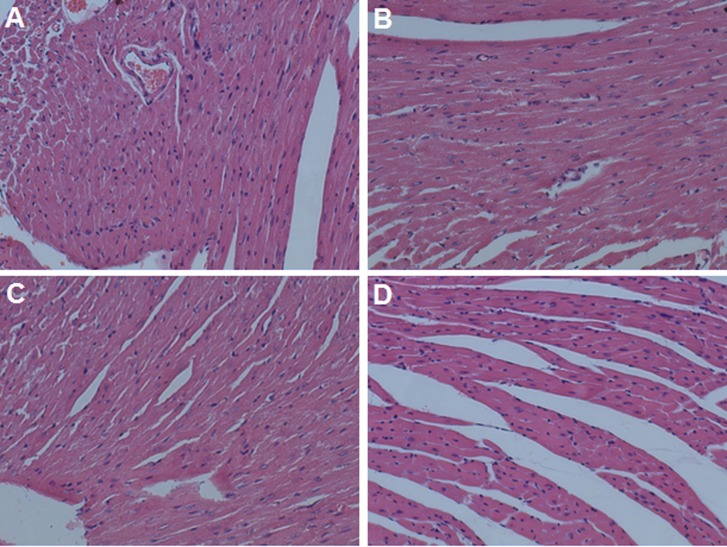
Hematoxylin and eosin (H&E) stained cardiac sections isolated from mice at 24 hours after the last intravenous dosing of different treatments. (A) Saline, (B) DOX+PTX, (C) PF-DP and (D) c(RGDyK)-FP-DP. Original magnification: ×20.

**Fig 9:**
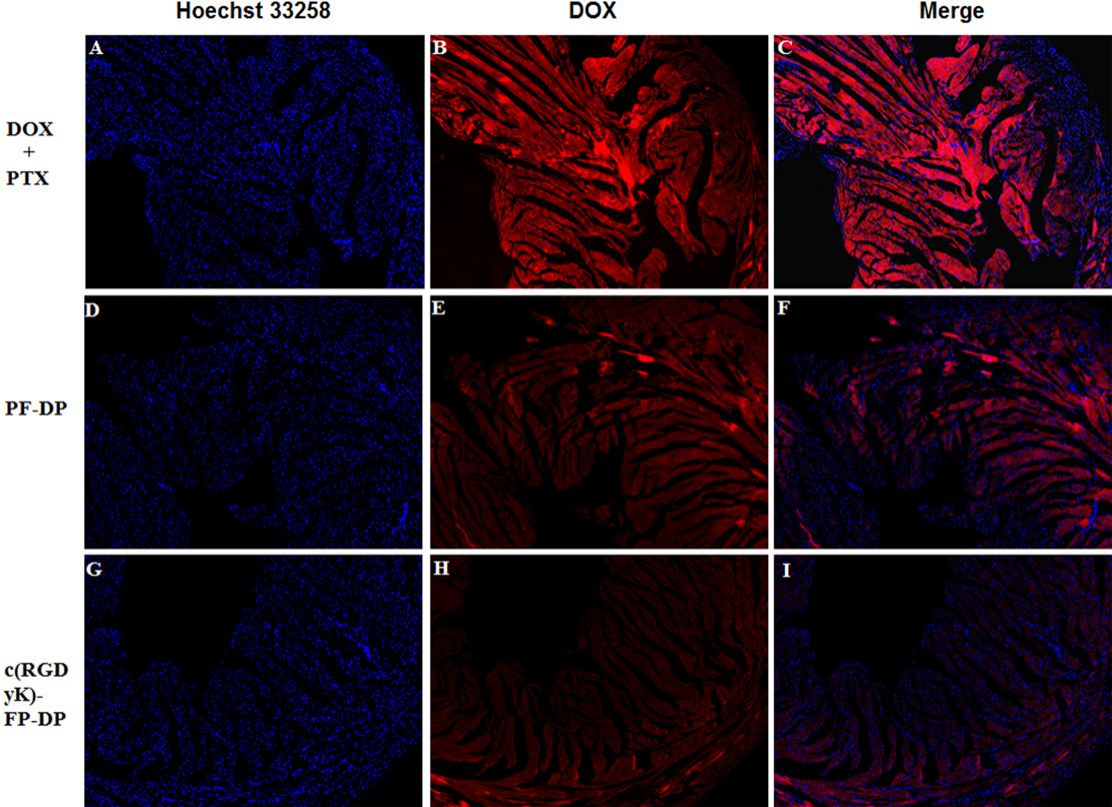
Fluorescence images of cardiac sections from KBv tumor-bearing BALB/c nude mice after systemic administration of different treatments. (A, B, C) DOX+PTX, (D, E, F), PF-DP or (G, H, I) c(RGDyK)-FP-DP. Red: DOX; Blue: Hoechst 33258. Original magnification: ×20.

Cardiotoxicity is one of the main side effects for anthracycline use, As reported, daily injection of DOX can cause weight loss and cardiotoxicity.[42] In our study, intravenous administration of free DOX and PTX mixed solution caused noticeable cardiac tissue degeneration, necrosis and heart congestion (Fig 8). The distribution of DOX in heart was further evaluated by fluorescent microscope. Results showed that DOX distribution of DOX+PTX group in heart was higher than those of micelle groups, and the red fluorescence of DOX in c(RGDyK)-FP-DP group was found to be less intense than PF-DP group (Fig 9). It was consistent with the results of biodistribution studies, suggesting less c(RGDyK)-FP-DP distributed in heart tissue. Besides, there was no heart muscle damage and no acute cardiotoxicity observed in PF-DP and c(RGDyK)-FP-DP group (Fig 8), suggesting that the cardiotoxicity of DOX can be alleviated when delivered by polymeric micelle system, probably due to a modified DOX biodistribution behavior conferred by the micellar nanocarrier system as evidenced in biodistribution study.

## 4. Conclusion

In this study, anticancer drug-loaded Pluronic-based polymeric micelles functionalized with c(RGDyK) peptide were tested as an efficient targeted vehicle for enhancing the treatment of KBv tumor. Compared with conventional PF-DP, c(RGDyK)-FP-DP displayed higher cellular uptake ability in KBv tumor cells and greater cytotoxicity *in vitro*. In addition, the *in vivo* tissue distribution evaluation demonstrated that c(RGDyK)-FP-DP could significantly increase the plasma and tumor accumulation of DOX and PTX, and decrease the cardiac accumulation of DOX compared with PF-DP. The anti-tumor efficacy of c(RGDyK)-FP-DP was also significantly enhanced in comparison with that of PF-DP and free drug. Safety evaluation showed no subacute toxicity to hematological system or heart post successive intravenous administration of c(RGDyK)-FP-DP.
